# A stress paradox: the dual role of the unfolded protein response in the placenta

**DOI:** 10.3389/fendo.2024.1525189

**Published:** 2024-12-20

**Authors:** Diba Chowdhury, Chloe E. Jang, Patrick Lajoie, Stephen J. Renaud

**Affiliations:** ^1^ Department of Anatomy and Cell Biology, Schulich School of Medicine and Dentistry, Western University, London, ON, Canada; ^2^ Department of Biochemistry, Schulich School of Medicine and Dentistry, Western University, London, ON, Canada; ^3^ Children’s Health Research Institute, Lawson Health Research Institute, London, ON, Canada

**Keywords:** ER stress, UPR, placenta, trophoblast, decidua

## Abstract

The placenta is a temporary organ that forms during pregnancy and is essential for fetal development and maternal health. As an endocrine organ, proper placental function requires continual production, folding, and transport of proteins and lipids. Central to these processes is the endoplasmic reticulum (ER), a dynamic organelle responsible for maintaining cellular protein and lipid synthesis and processing. ER stress occurs when there is an accumulation of unfolded or misfolded proteins, which triggers the activation of cellular pathways collectively called the unfolded protein response. Unfolded protein response pathways act to alleviate the misfolded protein burden and restore ER homeostasis, or if unresolved, initiate cell death. While prolonged ER stress has been linked to deficient placental function and adverse pregnancy outcomes, basal activation of unfolded protein response pathways is required for placental development and function. This review explores the importance of ER homeostasis in placental development and function, examining how disruptions in ER stress responses may contribute to adverse pregnancy outcomes.

## 1 Introduction

The endoplasmic reticulum (ER) is a membrane-bound organelle that plays a central role in the synthesis, folding, and transport of proteins, as well as lipid production and calcium storage. It is a dynamic structure, adapting and remodeling itself in response to cellular demands. This adaptability is critical for maintaining cellular proteostasis, thereby balancing protein synthesis, folding, and degradation. ER stress occurs when the capacity of the ER to properly fold proteins is overwhelmed, leading to an accumulation of misfolded or unfolded proteins in the ER. ER stress triggers the activation of cellular signaling pathways designed to take corrective actions and restore proteostasis, or to initiate cell death pathways if the burden is prolonged or severe. While ER stress and downstream signaling can occur in any cell, those with a high demand for protein synthesis such as hormone-producing cells are particularly susceptible. The placenta is one such organ tasked with producing numerous hormones and other proteins critical for fetal development and pregnancy success, where disruptions in proteostasis can have severe consequences for maternal and fetal health. In this review, we will present the importance of ER homeostasis for placental development and function, and discuss evidence linking ER stress with deficient placentation and adverse pregnancy outcomes.

## 2 The placenta

The placenta is a transient yet highly sophisticated organ that intimately connects the gestational parent and fetus. It forms the primary interface separating maternal and fetal tissue and facilitates metabolic exchange, endocrine functions, and fetal protection. The placenta regulates the exchange of nutrients, oxygen, carbon dioxide, and other substances between maternal and fetal circulations, while restricting the transfer of pathogens, xenobiotics, maternal immune cells and many other potentially harmful substances from accessing fetal blood (1, 2). The placenta is also an adaptive organ that can integrate information on maternal nutrient availability and fetal demands, and undergo dynamic morphological and molecular changes to support fetal development (3). As an endocrine organ, the placenta produces a plethora of hormones and other factors that regulate maternal adaptations to pregnancy along with fetal growth and development (4, 5).

## 3 An overview of placental structure

**Figure 1:**
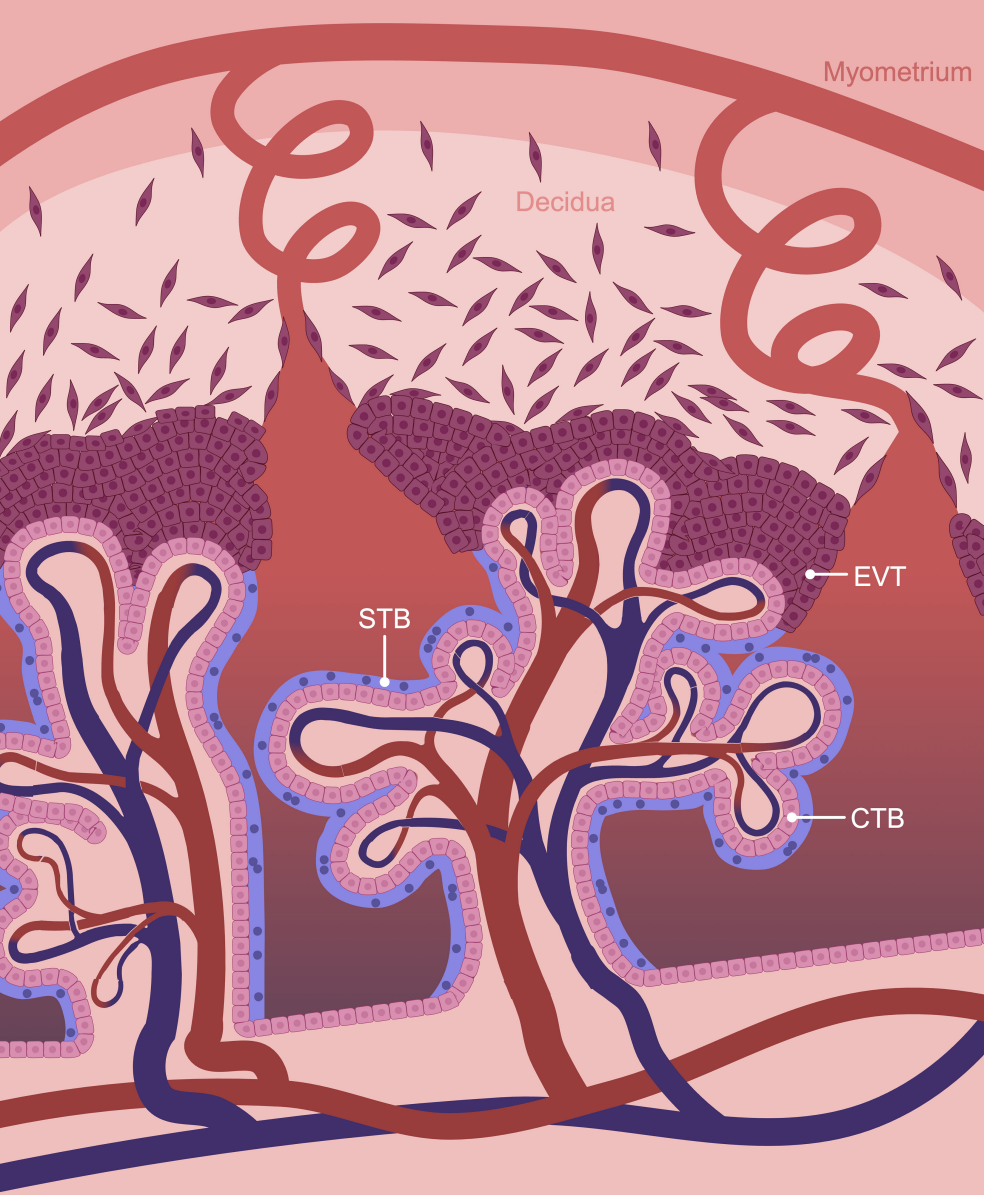
Schematic representation of the maternal-fetal interface. The human placenta is composed of chorionic villi which are lined by a trophoblast bilayer containing an inner cytotrophoblast (CTB) and outer syncytiotrophoblast (STB) layer. Extravillous trophoblasts (EVTs) form the cell columns at the tips of anchoring villi adjacent to the decidua and are the source of invasive trophoblasts. Created in BioRender. Chowdhury, D. (2024) https://BioRender.com/f22l744.

The human placenta is arranged into highly branched tree-like villi containing an inner core of macrophages, fibroblasts, pericytes, connective tissue, and capillaries that connect to the fetal circulation through the umbilical vessels. The villous core is separated from maternal blood by a trophoblast bilayer: an inner portion of cytotrophoblasts (CTBs) resting on a basement membrane, and an outer syncytiotrophoblast (STB) layer (**Figure 1**). CTBs are self-renewing progenitor cells that initially form a continuous layer during early pregnancy, and replenish the STB layer by fusing into it. In late pregnancy, the proportion of CTBs dwindles and they form a disjointed layer beneath the STB. The STB layer, on the other hand, is a multinucleated entity with a vast interconnected cytoplasm that lines the placental villi and bathes in maternal blood. It forms the primary boundary between maternal blood and fetal tissue, and is responsible for numerous placental functions including maternal-fetal gas and nutrient exchange (6, 7). Given its proximity to maternal blood, STB is responsible for the production and metabolism of numerous hormones, such as human chorionic gonadotropin (hCG). These factors are deposited into maternal circulation to alter maternal physiology and metabolism for fetal benefit. Furthermore, since the STB does not contain intercellular junctions, it forms a semi-exclusive barrier that restricts the passage of many substances from accessing fetal circulation (8).

At sites where large anchoring villi contact the decidua basalis (the specialized endometrial tissue adjacent to where the placenta forms), CTBs differentiate into a distinct cell-type: extravillous trophoblasts (EVTs). Proximally, EVTs proliferate into stratified cellular columns. At the distal tips of these columns, cells stop replicating and gain invasive properties. Invasive EVTs infiltrate into the decidua basalis and inner third of the myometrium. EVTs are versatile cells that affix the placenta to the decidua basalis, interact with decidual stromal cells and immune cells to support immunological tolerance, and remodel uterine blood vessels and glands to ensure that a consistent supply of nutrients and oxygen are delivered to the placenta to support fetal sustenance (9).

The decidua basalis is a dynamic tissue derived from the endometrium adjacent to where the placenta forms. Transformation of the endometrium into the decidua begins during the secretory phase of the menstrual cycle and accelerates upon fertilization. Decidualization involves the differentiation of fibroblast-like endometrial stromal cells (ESCs) into polygonal decidual stromal cells (DSCs) along with extensive development of uterine glands and blood vessels, processes that are tightly regulated by estrogen and progesterone (10). Transformation of ESCs into DSCs is associated with a substantial increase in the metabolic demand and secretory activity of the cells that serve essential roles in early embryo nutrition and maternal-fetal communication (11).

Placental structure exhibits marked diversity between species. In humans and closely-related primates, the placenta is termed “hemochorial” since maternal blood directly contacts trophoblasts. Other species with hemochorial placentation include common laboratory rodents like mice, rats, and guinea pigs. Although these species exhibit notable differences in placental anatomy and physiology compared to humans, there are also many similarities. For example, placentas of mice, rats, and guinea pigs have a labyrinth zone containing syncytialized trophoblasts that specialize in nutrient and gas exchange (akin to the placental villi in humans), and a junctional zone adjacent to the decidua basalis that anchors the placenta to the decidua and is the site where invasive trophoblasts emanate (analogous to human EVTs). Consequently, rodents are often used as laboratory models to gain deeper mechanistic insight into placental development and maternal-placental-fetal interactions (12). Although this review will focus mostly on placentation in humans, much knowledge has been derived from studies using rodent models and these studies will be mentioned when appropriate.

## 4 Placenta-associated pregnancy complications

Deficient placental development and function is a major culprit underlying severe pregnancy complications that compromise maternal and fetal well-being and survival. For example, inadequate placental development resulting in placental insufficiency (inadequate maternal blood supply to the placenta) can lead to preeclampsia—a common and dangerous pregnancy disorder characterized by sudden-onset maternal hypertension, endothelial dysfunction, and organ damage. In many cases, in preeclampsia the fetus does not obtain adequate oxygen and nutrients, resulting in fetal growth restriction (FGR) and the potential for long-term health deficiencies. Placentas from preeclampsia and FGR often exhibit evidence of hypoxia, oxidative stress and inflammation—conditions that can adversely affect ER homeostasis and result in ER stress (13–15). In this review, we will discuss ER homeostatic mechanisms required for placental development and function, and describe how these mechanisms are perturbed following cellular exposure to stress during placenta-associated pregnancy complications.

## 5 ER stress

Protein turnover in the placenta increases throughout pregnancy, requiring efficient quality control mechanisms to ensure that the protein folding capacity of the ER meets protein synthesis demands (16, 17). Key components of this quality control machinery include ER-resident chaperones such as binding immunoglobulin protein (BiP; also called glucose regulated protein 78, GRP78), calreticulin, calnexin, and protein disulfide isomerases (PDIs). These chaperones assist proteins in achieving their native conformation (18). However, if chaperones cannot keep pace with protein folding demands, an accumulation of unfolded or misfolded proteins in the ER lumen can result, leading to ER stress and the activation of the unfolded protein response (UPR) (19). The role of ER-associated chaperones for placentation and decidualization are summarized in **Supplementary Table 1**.

ER stress can occur due to an accumulation of abnormal lipids or misfolded proteins in the ER (20, 21). The accumulation and aggregation of misfolded proteins can occur due to increased rates of protein synthesis, deficiency in autophagy, nutrient deprivation, and dysregulated calcium levels (22, 23). Many cell types experience a high protein load during various stages of differentiation and maturation, resulting in ER stress. Induction of ER stress can have morphological and biochemical consequences to cells. Morphologically, ER stress can result in dilation of ER cisternae to increase the lumen size and accommodate the sudden increase in protein accumulation (24, 25). Many cells that experience ER stress undergo a degree of epithelial-to-mesenchymal transition, including increased N-cadherin levels and decreased E-cadherin levels (26). Biochemically, ER stress can activate several signaling pathways including the UPR, in an effort to resolve the stress and restore proteostasis (27).

Recent studies have characterized early-onset preeclampsia as a proteinopathy due to presence of misfolded proteins in maternal urine and serum (28–30). Misfolded oligomeric proteins and amyloids, arising from defective chaperone function or impaired autophagy, may either deposit in the placenta from maternal circulation or be produced directly by placental cells (31). Several amyloidogenic proteins, including amyloid-β, transthyretin, and α-1-antitrypsin, have been identified in the preeclampsia-associated misfoldome and proposed as potential diagnostic markers for preeclampsia (31–33). Deposition of protein aggregates in the placenta may induce cellular toxicity, impair nutrient and gas exchange, and lead to placental insufficiency. Exposure of placental explants to serum from preeclamptic pregnancies induces ER stress and the formation of syncytial knots, suggesting that maternal serum in preeclampsia may contain ER stress-inducing factors that promote protein aggregation in placental cells (34, 35). While the placenta can efflux misfolded proteins into maternal blood, the accumulation of protein aggregates in maternal blood may pose risks for maternal organs (31). Therefore, targeting of misfolded proteins and resolution of ER stress could be promising therapeutic strategies to restore cellular homeostasis and improve pregnancy outcomes (36–38).

## 6 The UPR in placentation

**Figure 2:**
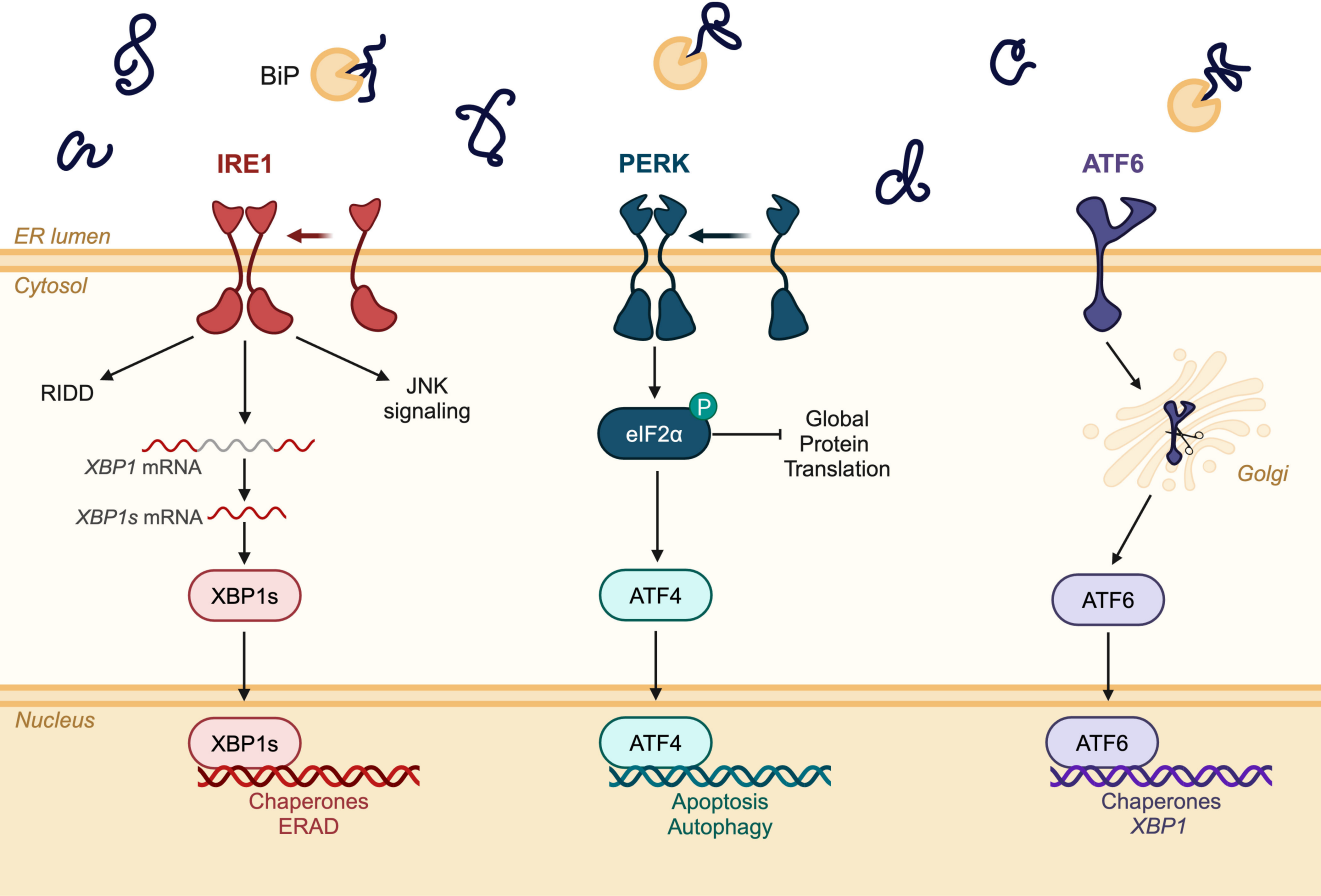
Schematic diagram of the three signaling branches of the unfolded protein response (UPR). Inositol-requiring enzyme 1 (lRE1); protein kinase R-like ER kinase (PERK); activating transcription factor 6 (ATF6); regulated IRE1-dependent decay (RIDD); jun N-terminal kinase (JNK). Created in BioRender. Chowdhury, D. (2024) https://BioRender.com/r03w626.

The UPR is a conserved cellular response to ER stress. In principle, the UPR seeks to correct the accumulation of unfolded and misfolded proteins in the ER, thereby restoring ER homeostasis. Basal levels of UPR activation promote cellular homeostasis, but in cases of prolonged or severe ER stress, unmitigated UPR activation can lead to cell death (39, 40). The UPR pathway consists of three ER-resident sensor proteins: inositol-requiring enzyme 1 (lRE1), protein kinase R-like ER kinase (PERK), and activating transcription factor 6 (ATF6), all of which trigger a transcriptional response to adapt the ER folding environment by upregulating genes involved in protein folding and degradation (**Figure 2**) (41). The following sections will summarize the role of IRE1, PERK, and ATF6 signaling in the context of placentation and decidualization.

## 7 IRE1

IRE1 is a transmembrane protein that is evolutionarily conserved from yeast to humans (42, 43). It contains a serine/threonine kinase domain and a cytosol-facing domain with endoribonuclease activity. There are two isoforms of IRE1 in mammals – IRE1α and IRE1β – with IRE1α being the ubiquitously expressed isoform (44). Upon detection of misfolded or unfolded proteins, IRE1 homodimerizes and activates its kinase function through trans-autophosphorylation of Ser^724^, Ser^726^, and Ser^729^ in the kinase activation loop (45). Once activated, IRE1 splices out a 26-nucleotide intron from the X-box binding protein 1 (*XBP1*) mRNA sequence, encoding for the spliced XBP1 (XBP1s) transcription factor (46). Thus, IRE1 activation is typically measured through the phosphorylation of IRE1, splicing of *XBP1* mRNA, or the accumulation and nuclear localization of the XBP1s transcription factor. XBP1s transcriptionally regulates genes encoding various chaperones, ER-associated degradation (ERAD) components, and proteins involved in lipid biosynthesis to relieve ER stress (47). The endoribonuclease activity of IRE1 can also degrade other mRNA sequences through regulated IRE1-dependent decay to reduce protein synthesis in the ER lumen (48, 49). While the activation of IRE1 is associated with cell survival mechanisms to relieve ER stress, persistent IRE1 activity can induce apoptosis by activating tumor necrosis factor receptor-associated factor 2, apoptosis signal-regulating kinase 1, and mitogen-activated protein kinases (26, 50).

### 7.1 IRE1 is required for placental development in mice

IRE1α (herein referred to as IRE1) is essential for placental development in mice. Embryos and placentas with a deletion in *Ern1*, the gene that encodes IRE1, are smaller than wild-type and heterozygous siblings, and die around embryonic day 12.5 (51–53). *Ern1^-/-^* placentas have reduced blood spaces in the labyrinth zone compared to wild-type mice, accompanied by decreased expression of vascular endothelial growth factor, suggesting that IRE1 contributes to placental vascular development (52–54). There does not appear to be an effect on decidualization in these mice (52). Interestingly, activation of IRE1 signaling, based on detecting IRE1 phosphorylation and *Xbp1* splicing, occurs in the placenta throughout early and midgestation but not in the embryo (52), suggesting that IRE1 signaling is particularly important for placental development. In line with this observation, midgestation lethality is avoided in mice lacking *Ern1* in embryonic tissue but not trophoblasts (using a *Mox2^+/Cre^* transgenic mouse), demonstrating that the cause of embryonic demise in *Ern1^-/-^* mice is due to defective placental development (52). Therefore, IRE1 appears to have a critical role for placental development in mice.

In line with an essential role of IRE1 signaling for placentation, high levels of XBP1s are detectable in the mouse placenta, but not in other embryonic organs (52). XBP1s is also spatiotemporally expressed in the mouse uterus where it is mainly detected in epithelial cells during early pregnancy and then subsequently in DSCs (55, 56). To investigate whether canonical IRE1-XBP1s signaling is involved in placental development, *Xbp1^-/-^* mice have been generated. Interestingly, embryos and placentas with a deletion in *Xbp1* exhibit embryonic lethality between embryonic day 12.5 and 14.5; however, unlike *Ern1* ^-/-^ mice, the cause of lethality is attributed to liver hypoplasia. There does not seem to be a notable difference in placental morphology or vascular endothelial growth factor levels between *Xbp1^-/-^* mice and wild-type mice (52, 57). Therefore, the essential role of IRE1 in mouse placental development may be through a noncanonical signaling pathway independent of XBP1s.

### 7.2 IRE1 in human STB formation

There is strong evidence that IRE1 signaling is active and required during human STB formation. Activation of IRE1 signaling may be downstream of cyclic adenosine monophosphate (cAMP)-protein kinase A (PKA) signaling, a well-established inducer of trophoblast syncytialization (58, 59). PKA phosphorylates IRE1 on Ser^724^ (58, 60), and also activates cAMP-response element binding protein, which is a transcriptional activator of *ERN1 (* 61). STB formation *in vitro* using primary CTBs from term placenta is associated with IRE1 activation. Similar results are observed using BeWo cells, a CTB-like choriocarcinoma line that fuses to form STB-like cells after exposure to forskolin (62). Interestingly, the addition of small molecule inhibitors targeting IRE1, or RNA-interference targeting all three UPR branches, reduces CTB fusion, hCG secretion, and autophagy (62). Therefore, similar to its important role in mouse placentation, IRE1 appears to be required for human STB formation.

Why is IRE1 signaling needed for STB formation? While this answer remains unclear, it may be related to the increased synthesis and secretion of numerous proteins involved in the differentiation of CTBs and their fusion into STBs. Increased demand for protein synthesis during STB formation can exceed the ER’s folding capacity, resulting in the activation of the UPR to adapt to the new protein burden. Since IRE1-XBP1s signaling promotes the production of chaperones and ERAD components, its activation during STB formation may promote protein folding and clearance to ensure proper proteostasis in the ER, thereby enabling STB function. IRE1 activation may also promote the expression of genes involved in phospholipid biogenesis to remodel the ER membrane and expand the ER lumen during STB formation (63, 64).

Apart from responding to ER stress signals, XBP1s transcriptionally regulates the expression of differentiation-associated genes in other cells (65, 66). It is therefore possible that XBP1s may exert transcriptional control of genes needed for STB formation and function, such as ER-resident proteins needed for protein processing of STB-related hormones. As one example, the prototypical STB hormone, hCG, is a glycoprotein containing 11 disulfide bonds. These disulfide bonds are essential for hCG to fold into its proper protein configuration (67–69). XBP1s transcriptionally regulates the expression of PDIs, which are needed in the ER for disulfide bond formation and stability of this protein (70). Furthermore, XBP1s also transcriptionally regulates secretory pathway components, including those involved in protein glycosylation and vesicle trafficking needed for maturation and secretion of hCG. Thus, IRE1-XBP1s signaling may be required for the synthesis, processing, and secretion of hCG, along with other STB-associated proteins (71).

CTB fusion involves dynamic changes in membrane composition and membrane-membrane interactions facilitated by syncytins, resulting in the formation of fusion pores, merging of membranes, and intermixing of cytoplasm between adjacent cells (72). While there is no evidence to-date that IRE1-XBP1s signaling transcriptionally regulates the expression of syncytins or their receptors, a role for IRE1-XBP1s signaling has been identified in other models of cell fusion. For example, deletion of IRE1 in mouse muscle satellite cells inhibits myoblast fusion and impairs skeletal muscle regeneration. IRE1-XBP1s signaling directly controls the expression of several genes involved in myoblast fusion, including *Mymk* (encoding Myomaker) (73). IRE1-XBP1s may similarly regulate the expression of genes involved in STB formation; or at minimum, may be needed to produce chaperones and other proteins that facilitate the synthesis, transport, and membrane presentation of fusogens.

Aside from direct transcriptional control of fusogens, IRE1 may play a role in cell fusion during STB formation by interacting with filamin A, an actin binding protein that regulates cytoskeletal organization. IRE1 acts as a scaffold by recruiting kinases, such as protein kinase C alpha, to promote phosphorylation of filamin A and induce cytoskeletal remodeling, an essential process that occurs during cell fusion (74–76). Furthermore, IRE1 may facilitate cell fusion by initiating contact sites between the ER and plasma membrane through store-operated calcium channels, which regulate intracellular calcium levels and maintain lipid homeostasis. IRE1 interacts with stromal interacting molecule 1 (STIM1), a sensor of calcium levels in the ER lumen (77). When calcium levels in the ER lumen are low, STIM1 undergoes a conformational change to promote calcium entry from the cytosol to the ER lumen (77, 78). Therefore, by interacting with STIM1, IRE1 may regulate cytosolic calcium levels, which along with increased cAMP, is a requirement for STB formation (79).

### 7.3 IRE1 in human EVT formation and function

IRE1 signaling is enriched in first trimester EVTs compared to CTBs, and gene set enrichment analysis of EVTs derived from human trophoblast stem cells shows enrichment of XBP1s-induced chaperones (80). Furthermore, the same study showed that inhibition of IRE1 using a small molecule inhibitor, 4µ8c, reduces cell surface HLA-G expression, suggesting that IRE1-XBP1s signaling is active during EVT formation and may be required for the synthesis and/or transport of cell surface antigens.

While the specific role of IRE1-mediated signaling in EVT formation and invasiveness is not fully understood, interactions with other factors deemed important for EVT function have been noted. For instance, Krüppel-like factor 6 (KLF6) is a transcription factor highly expressed in the placenta that modulates EVT formation and invasiveness. Using HTR-8/SVneo cells, an immortalized cell-line derived from first trimester placental outgrowths and often used as a model of invasive EVTs, silencing KLF6 reduces IRE1 and XBP1s levels, suggesting that KLF6 may be upstream of IRE1 signaling. Interestingly, both KLF6 and IRE1 deficient mouse models are embryonic lethal at embryonic day 12.5, suggesting that they may be part of a similar developmental pathway (52, 81). IRE1 may also promote the expression of high-temperature requirement A serine peptidase 1 (HTRA1) following exposure to ER stress conditions, which promotes invasion of HTR-8/SVneo cells (82, 83). Notably, in other cells, IRE1-XBP1s signaling promotes the transcription of genes encoding factors associated with epithelial-to-mesenchymal transition, such as *SNAI1*, *SNAI2*, *ZEB2* and *TCF3 (* 84). EVTs undergo epithelial-to-mesenchymal transition as they gain invasive properties (85), but further studies are needed to determine whether IRE1-XBP1s signaling may contribute to this aspect of EVT development.

### 7.4 IRE1 signaling during human decidualization

There is evidence that IRE1-XBP1 signaling is active during decidualization. For example, cytoplasmic and nuclear reactivity for unspliced and spliced variants of XBP1 are detected in both DSCs and glandular epithelium (86). Decidualization of ESCs is associated with increased levels of IRE1 and its downstream targets, XBP1s and CCAAT/enhancer-binding protein homologous protein (CHOP). Treatment of DSCs with the IRE1 inhibitor, STF-083010, downregulates the expression of CHOP and prevents the secretion of interleukin-1β (IL-1β) associated with decidualization (87). Furthermore, in a co-culture model designed to mimic implantation, inhibition of IRE1 reduced invasion of trophoblast spheroids (derived from Swan-71 immortalized trophoblasts) into DSCs, suggesting that IRE1 may be required for this process (87). While IRE1-mediated signaling may be required for decidualization, hyperactivation may induce autophagy and disrupt decidualization. For example, the IRE1-XBP1s pathway is negatively regulated by the heat shock cognate 71 kDa (Hsc70). Knockdown of Hsc70 increases XBP1 protein expression, triggers autophagy, and impairs decidualization of ESCs (88). These results suggest that moderate activation of IRE1 supports successful decidualization, whereas too little or too much activation can interfere with this process.

### 7.5 IRE1 signaling in the placenta during pregnancy complications

Aberrant ER stress and hyperactivation of IRE1 signaling is associated with dysregulated placental development and adverse pregnancy outcomes. This has been most frequently documented in pregnancies complicated by preeclampsia and FGR. Compared to placentas from normotensive pregnancies, placentas from preeclamptic pregnancies (particularly early-onset preeclampsia) exhibit increased IRE1 phosphorylation, XBP1 splicing, and ER luminal dilation (89, 90). Likewise, expression of *XBP1* is increased in decidual tissues from preeclampsia with and without FGR (86). Endometrial biopsies from patients with recurrent pregnancy loss and implantation failure have elevated expression of IRE1 compared to controls, but surprisingly lower expression of XBP1s, suggesting that dysregulated IRE1 activation may contribute to these pregnancy complications (87). However, phosphorylated IRE1 was not measured in the samples from recurrent pregnancy loss and implantation failure, and should be evaluated in future studies to determine whether IRE1 activity may be altered in these pregnancy complications.

IRE1 phosphorylation, along with other markers of ER stress and apoptosis, is also increased when subjecting trophoblasts to hypoxia-reoxygenation *in vitro* to mimic the conditions that placental cells would experience in early-onset preeclampsia (91–93). Therefore, IRE1 signaling likely contributes to the cellular stress response during pathological conditions. Other pregnancy complications associated with ER stress and IRE1 activation include gestational cholestasis, a condition in which bile acids build up in the liver and enter the bloodstream. In a mouse model of gestational cholestasis, increased IRE1 activation is apparent in placental tissue, and this was associated with apoptosis and FGR. Inhibition of IRE1 signaling using 4µ8c prevents trophoblast apoptosis and rescues FGR in mice. Likewise, inhibiting IRE1 signaling prevents cell death in the HTR-8/SVneo cell-line following exposure to deoxycholic acid (94).

Physiological and environmental stressors that increase the risk of pregnancy complications can induce ER stress and activate IRE1 signaling. For example, maternal obesity is a risk factor for various pregnancy complications. Compared to placentas from normal weight pregnancies, obese pregnancies exhibit increased levels of phosphorylated IRE1 and XBP1s in placental tissue (95, 96). Palmitate is the most common saturated fatty acid in the body and is detectable at higher levels in the circulation of obese persons. Increased levels of palmitate alter ER morphology, impair invasiveness, and induce ER stress and apoptotic signaling in various human trophoblast cell-lines (97, 98). Other environmental stressors associated with ER stress and IRE1 hyperactivation in trophoblasts include viral infections (e.g., ZIKV) and exposure to toxins such as nicotine and ethanol (99–102). In the latter case, only total IRE1 protein levels were assessed, and therefore the contribution of active IRE1-XBP1s signaling to placental function following exposure to toxins remains elusive.

## 8 PERK

PERK detects and responds to ER stress by reducing the amount of protein in the ER lumen through the attenuation of translation (103). When there is an overaccumulation of misfolded proteins in the ER lumen, PERK homodimerizes and trans-autophosphorylates at Thr^980^, resulting in the activation of its kinase function (104). PERK will subsequently phosphorylate eukaryotic initiation factor 2α (eIF2α), preventing the assembly of ribosomes at the initiator codon of mRNA transcripts and blocking protein synthesis (105). As part of the PERK signaling branch, phosphorylated eIF2α promotes mRNA translation of the activating transcription factor 4 (ATF4), which can modulate transcription depending on its binding partners. The classical transcriptional target of ATF4 during ER stress is *DDIT3*, which encodes for CHOP, a transcription factor that regulates apoptosis (106). ATF4 can also transcriptionally regulate genes involved in amino acid metabolism and autophagy (107).

### 8.1 PERK regulates proper protein folding in the mouse placenta

Mice lacking PERK are viable and do not initially differ in weight compared to their wild-type counterparts, suggesting that PERK activity is not required (or is redundant) for placental and embryo development (108). However, other organs with secretory activity are severely impacted by PERK deficiency. For example, mice lacking PERK exhibit progressive loss of pancreatic β cells, neonatal development of diabetes mellitus, and exocrine pancreatic atrophy. Pancreatic cells in these mice have ER lumen dilation and accumulation of electron-dense material in the ER. Mice lacking PERK also have severe skeletal dysplasias and dwarfism associated with reduced hepatic secretion of insulin-like growth factor 1 (108–110). Since the placenta is also a secretory organ, PERK deficiency may affect aspects of placental endocrine function.

To determine a potential contribution of PERK signaling to placental endocrine activity, a conditional mouse model with PERK depletion specifically in the junctional zone has been investigated. The junctional zone secretes numerous hormones and growth factors, and is therefore considered as the endocrine component of the murine placenta (111). There are no differences in litter size, placental weight or fetal weight between wild-type mice and those with junctional zone-specific PERK depletion (as expected, since the global PERK knockout also does not show these differences). However, PERK deficiency exacerbates ER stress in the junctional zone when mice are housed in a reduced oxygen atmosphere, which was done to induce tissue hypoxia and ER stress. When exposed to hypoxia, PERK-deficient junctional zone trophoblasts exhibit dilation of the ER cisternae and accumulation of protein aggregates, indicating possible loss of ER homeostasis through aggregation of misglycosylated secretory proteins in the ER (111, 112). Furthermore, these trophoblasts have a reduced capacity to stimulate maternal physiological adaptions, such as the induction of glycogenolysis in the liver. Therefore, in the placenta, PERK may promote proper protein folding and processing in the ER, whereas PERK-deficient mice may be more susceptible to proteinopathies under ER stress-inducing conditions.

Activation of PERK attenuates translation through the phosphorylation of eIF2α on Ser^51^ (113). A mouse model has been generated in which this serine is mutated to alanine, preventing the ability of eIF2α to reduce translation (114). These mice die shortly after birth due to hypoglycemia, resulting from a deficiency in gluconeogenesis and loss of pancreatic β cells. In the placenta, eIF2α mutant mice exhibit increased basal translation, correlating with reduced placental and fetal growth compared to mice possessing at least one wild-type copy (112). Additionally, these placentas have an accumulation of glycoproteins in the junctional zone and reduced labyrinth zone volume, suggesting that eIF2α dysfunction may disrupt placental endocrine function (112).

### 8.2 PERK signaling in human placental and decidual development

As a key regulator of protein translation, PERK signaling may contribute to the control of trophoblast differentiation. For example, PERK signaling is activated in BeWo cells during forskolin-induced differentiation as shown through increased levels of phosphorylated eIF2α, ATF4, and CHOP. Exposing BeWo cells to a PERK inhibitor, GSK2656157, inhibits cell fusion, but interestingly there is no effect on secreted hCG levels. Similar results are obtained using primary CTBs from term placentas (62). Like IRE1, PERK may promote trophoblast fusion through its interaction with filamin A to regulate cytoskeletal remodeling and ER calcium levels (115). In HTR-8/SVneo cells and JEG-3 cells, another transformed trophoblast cell-line, induction of ER stress using tunicamycin, thapsigargin, or pro-inflammatory cytokines reduces matrix metalloproteinase 2 (MMP2) levels and cell invasion. However, inhibition of PERK restores MMP2 levels (116). Given the importance of PERK signaling for ER homeostasis in other secretory cells, PERK may maintain cellular integrity in the placenta, particularly under ER stress-inducing conditions.

Within the decidua, DSCs from pregnancies deemed healthy express high levels of phosphorylated PERK. Treatment of ESCs and glandular cells with progesterone activates the PERK pathway, as shown by the increased levels of phosphorylated eIF2α, ATF4, and CHOP (87, 90, 117). Progesterone-driven CHOP expression induces several apoptosis markers (e.g., BAX and cleaved caspase-3) in ESCs and glandular cells. Furthermore, BiP/GRP78, CHOP, and cleaved caspase-3 are upregulated during the secretory phase in stromal and glandular regions of healthy human endometrium, confirming that UPR activation and CHOP-mediated apoptosis increase in response to higher progesterone levels (117). Altogether, these findings indicate that progesterone-driven activation of the PERK/eIF2α/ATF4 pathway, and subsequent CHOP-mediated apoptosis, may regulate endometrial remodeling during the late secretory phase.

### 8.3 PERK signaling in the placenta during pregnancy complications

PERK signaling may have important roles in determining cell function and cell fate during placental pathologies. For example, in placentas from preeclamptic pregnancies, increased levels of phosphorylated PERK, phosphorylated eIF2α, ATF4 and CHOP are evident, and these proteins appear to localize predominantly to the STB layer (118, 119). Increased levels of phosphorylated PERK are also evident in the decidua (90). Decidual tissue from preeclamptic pregnancies with FGR have increased phosphorylation of eIF2α and ATF4 levels compared to control pregnancies (86, 120). These findings suggest that dysregulation of PERK signaling in the decidua may impair decidualization and contribute to the development of pregnancy complications.

Maternal serum from preeclamptic pregnancies activates the PERK pathway in placental explants and HTR-8/SVneo cells, as shown through increased eIF2α phosphorylation and CHOP levels. Cell death was also apparent after explants and cells were exposed to serum from preeclamptic pregnancies, but the specific contribution of PERK signaling to cytotoxicity was not assessed (34). It is possible that the PERK-mediated cell death observed in placentas from preeclamptic pregnancies occurs as a result of histone deacetylase (HDAC) deficiency (121). HDACs are essential for trophoblast differentiation and are often downregulated in placentas from preeclamptic pregnancies (121–124). HDAC2 silencing in HTR-8/SVneo cells increases pyroptosis, which was prevented when PERK was knocked down (121). Therefore, PERK activation may underlie the increased incidence of cell death in placental pathologies.

To recapitulate the ER stress-inducing conditions that may induce PERK signaling and affect trophoblast function and viability, several *in vitro* models of cell stress have been devised. For example, exposure of BeWo cells to hypoxia/reoxygenation (fluctuating between room air and 1% O_2_) leads to increased levels of phosphorylated eIF2α levels and reduced cell number (39). IL-1β, a pro-inflammatory cytokine that is increased in preeclampsia, induces apoptosis in BeWo cells through the PERK pathway, which is inhibited by progesterone (118). PERK inhibition can also reduce apoptosis in BeWo cells treated with endocannabinoid 2-arachidonoylglycerol (125). Cadmium, an environmental pollutant and carcinogen, reduces 11β-HSD2 expression in JEG-3 cells, which is restored either by knocking down PERK expression or by treating cells with antioxidants such as melatonin or N-acetylcysteine (126, 127). Thus, signaling through PERK may regulate trophoblast survival under various ER stress-inducing conditions.

## 9 ATF6

ATF6 is the third ER transmembrane sensor that is activated in response to ER stress (128). There are two isoforms of ATF6: ATF6α and ATF6β. Signaling through ATF6α generally results in a more potent but transient transcriptional response, whereas ATF6β is a comparatively weaker transcriptional modulator (129). During ER stress, the cytosolic domain of ATF6 translocates to the Golgi apparatus to be cleaved by site 1 and site 2 proteases (130). These proteases remove the luminal and transmembrane portions of ATF6, yielding the ATF6 transcription factor. ATF6 binds to the ER stress response element motif to transcriptionally regulate chaperones and ERAD components that enhance protein folding and contribute to clearance of misfolded proteins, respectively (131, 132).

### 9.1 ATF6 in mouse placental and decidual development

While various aspects of the UPR have been studied in the context of placental development, the role of ATF6 remains largely unexplored, highlighting a gap in understanding its potential contributions to trophoblast function and placental health. Knockout of either ATF6α or ATF6β in mice produces viable progeny, but double ATF6α/β knockout mice die early during development (around the time of implantation), suggesting that ATF6α and ATF6β may share an overlapping function that is essential for decidualization and early embryonic development in mice (133, 134). In the mouse uterus, expression of ATF6α is primarily localized to luminal and glandular epithelial cells, specifically near the blastocyst implantation site, suggesting a potential role in uterine receptivity (135). Since ATF6α is also present in primary and secondary decidualization zones, it is plausible that ATF6α could contribute to the progression of decidualization. Indeed, artificial decidualization of pseudopregnant mice increases ATF6α expression in decidual cells (135). Altogether, increased ATF6α levels in the uterus seem to correlate with the implantation window and initial stages of decidualization in mice, but whether ATF6α directly contributes to these processes requires further exploration.

### 9.2 ATF6 signaling in human placental and decidual development

ATF6α is present in the human placenta, and staining is particularly strong in nuclei clustered in syncytial knots (90). Exposure of primary CTBs to an ATF6α inhibitor, AEBSF, reduces cell fusion and hCG secretion (62, 136). It is possible that ATF6α promotes STB formation by regulating expression of BiP/GRP78 (137). Additionally, since ATF6α transcriptionally regulates *XBP1*, there may be contributory or compensatory mechanisms through which ATF6α influences STB formation by supporting the IRE1-XBP1s axis (131). ATF6α is also detected in EVTs, suggesting its participation in the invasion of EVTs into the decidua and interactions with decidual cells (86). In cancer cells, ATF6α promotes cell invasion and metastasis (138). Therefore, it is possible that ATF6α may regulate genes involved in EVT migration and invasion, but this requires further investigation.

Decidual cells from healthy pregnancies exhibit high levels of ATF6α localized primarily to the cytoplasm, indicating the presence of primarily inactive forms of ATF6α (90). Decidualization of ESCs is associated with an upregulation of ATF6α. Inhibition of ATF6 using AEBSF reduces the decidualization-associated increase in IL-1β secretion, which was also reported when using an IRE1 inhibitor (87, 139). It is unclear whether the mechanistic control of inflammatory cytokine production by ATF6α overlaps with the IRE1 signaling pathway.

### 9.3 ATF6 signaling in the placenta during pregnancy complications

In early-onset preeclampsia with FGR, ATF6α protein levels are increased in placental tissue compared to placentas from pregnancies deemed healthy (39, 86). It is unclear if ATF6α activation is also increased, as the levels of active ATF6α, or nuclear localization of the protein, were not assessed. A hallmark of placentas from preeclampsia is reduced secretion of placental growth factor, which is a pro-angiogenic molecule important for maintaining endothelial integrity. Interestingly, reduced placental growth factor in preeclampsia is associated with nuclear localization of ATF4 and ATF6β (but not ATF6⍺ or XBP1) in the STB layer (140, 141). Combined ATF4 and ATF6β silencing in BeWo cells exposed to the ER stressor thapsigargin or hypoxia/reoxygenation increased expression of placental growth factor (141), suggesting that ATF4 and ATF6β negatively regulate placental growth factor expression. Therefore, interfering with ATF4 and ATF6β may be a feasible means to enhance placental growth factor production in compromised pregnancies.

## 10 Future applications: targeting ER stress to alleviate placental dysfunction

As highlighted in the preceding sections, placental development and function require a fine balance of ER stress and UPR activation to maintain cellular homeostasis. Too much or too little UPR activation can impair placental and decidual function and lead to adverse pregnancy outcomes. Therefore, therapeutic approaches targeting ER stress pathways may be promising avenues to restore proper placental function and improve fetal and maternal outcomes (34, 142). As one example, tauroursodeoxycholic acid (TUDCA), a bile acid derivative that is currently being evaluated for the treatment for neurodegeneration, alleviates ER stress by acting as a chemical chaperone and reducing the expression of ER stress-related proteins (143). In a rat model of advanced maternal age, placental insufficiency accompanied by increased ER stress markers (p-eIF2α and CHOP) was observed in aged, pregnant rats. TUDCA treatment administered via drinking water throughout pregnancy reduced ER stress in the placenta and improved placental blood flow and fetal growth (38, 144–146). TUDCA treatment of pre-implantation embryos can increase the rate of blastocyst formation and enhance the success of implantation and pregnancy in mice (147). Another example includes phenylbutyric acid, a fatty acid that is naturally produced through fermentation by colonic bacteria, which ameliorates ER stress by acting as a chemical chaperone (148). Phenylbutyric acid reduces high glucose-induced ER stress in BeWo cells, suggesting that this compound may be useful to reduce placental ER stress in pregnancies with gestational diabetes (149). Additionally, phenylbutyric acid can also reduce hypoxia-induced apoptosis in HTR-8/SVneo cells by assisting in protein folding and reducing PERK activation (150). Overall, the use of chemical chaperones to facilitate protein folding and prevent UPR hyperactivation may be a promising avenue to treat placental pathologies and improve fetal outcomes.

Future studies could also explore whether selectively targeting one or more UPR pathways is an effective way to improve placental function (39, 151, 152). There are several small molecule inhibitors that specifically target these pathways and are being tested in humans for their efficacy in disease settings like cancer (153–155), but it remains uncertain whether these inhibitors can be safely used during pregnancy. Unintended off-target effects and safe delivery strategies need to be considered. Conversely, since UPR pathways are essential for various aspects of placental and decidual function, it is possible that transiently stimulating one or more pathways may be beneficial to restore ER homeostasis and promote cell survival. More research is required to determine whether ER stress-related pathways can be safely modulated to fine-tune placental ER homeostasis.

## 11 Conclusion and perspectives

**Figure 3:**
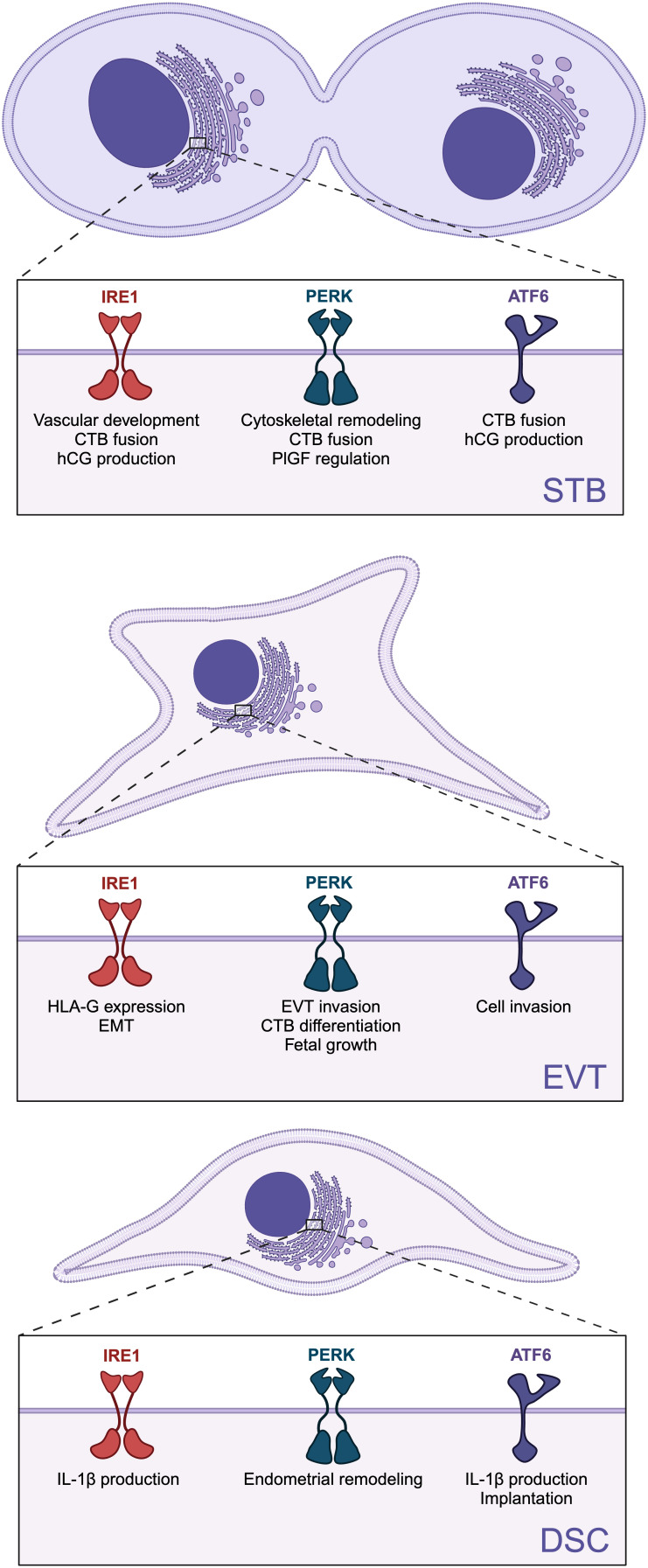
Role of specific unfolded protein response (UPR) branches on placental and decidual development and function. The three UPR sensors: inositol-requiring enzyme 1 (lRE1), protein kinase R-like ER kinase (PERK), and activating transcription factor 6 (ATF6) are involved in the differentiation and function of syncytiotrophoblast (STB), extravillous trophoblasts (EVTs), and decidual stromal cells (DSCs). Created in BioRender. Chowdhury, D. (2024) https://BioRender.com/x70x431.

In this review, we highlighted the importance of ER homeostasis and UPR signaling for placental and decidual development and function. Although there is resounding evidence of increased ER stress and UPR hyperactivation during pregnancy pathologies, each UPR signaling branch contributes to basic processes needed for placental and decidual function, and these functions may shift depending on cellular conditions (**Figure 3**). Therefore, tweaking the activity of UPR branches to restore ER homeostasis holds promise as a treatment approach to improve placental function in compromised pregnancies.

Despite significant advances in understanding the role of UPR pathways in placental and decidual development, several gaps remain. It is apparent that UPR functions extend beyond merely responding to stress, as UPR sensors participate in homeostatic mechanisms to facilitate the high demand for protein synthesis and processing in secretory cells. However, the precise roles of each UPR branch in placental and decidual functions are still elusive. Determining their specific contributions can be challenging because there is significant overlap and compensation between the branches and other stress-related pathways. For instance, *Ern1-*deficient mouse placentas exhibit increased PERK and ATF6 activation, which may mask additional ways that IRE1 regulates placental development and function (52). As another example, kinases associated with the integrated stress response, which are not connected to the UPR, can activate eIF2α independently of PERK (156). Therefore, phosphorylation of eIF2α does not necessarily guarantee that the PERK branch of the UPR is active. Additionally, the source of ER stress and UPR activation during placental and decidual development remains elusive. The high demand for protein and lipid production could be contributing to ER stress and UPR activation during placental and decidual development.

Another challenge with assessing ER stress, particularly in the context of placentation, is the limited tools that are available to confirm UPR branch activation. Many studies rely on detecting a handful of changes in gene expression or protein levels of UPR markers in bulk tissue or cell-lines. As the field evolves and better cell models and tools are developed, a more comprehensive understanding of each UPR branch is likely to emerge. For instance, the use of more complex cellular models of the decidua and placenta, such as decidual and trophoblast organoid cultures or “on-a-chip” platforms, offer promising avenues for better representing cellular interactions at the maternal-fetal interface. The use of these models in combination with more precise detection of UPR activation, such as reporter plasmids to measure activation of specific UPR branches in combination with omics technologies for more comprehensive coverage of downstream UPR targets, may provide a deeper understanding of the cellular response to stress at the maternal-fetal interface. The use of CRISPR-mediated gene targeting to disrupt each ER sensor in trophoblast and decidual models will also be enlightening. Collectively, uncovering the role of UPR branches holds potential for a greater understanding of ER homeostasis during normal and pathological placentation.
