# Supplementary material for: A stress paradox: the dual role of the unfolded protein response in the placenta
Source: Front Endocrinol (Lausanne). 2024 Dec 20;15:1525189. doi: 10.3389/fendo.2024.1525189 (PMC11695235; doi:10.3389/fendo.2024.1525189)
Supplement: Supplementary file 1 [file Table1.docx]

Supplementary Material

# Supplementary Figures and Tables

**Supplemental Table 1. Role of ER-resident chaperones in placental and decidual development.**

| **Chaperone** | **Condition/ Model** | **Function/Effect** | **Reference(s)** |
| --- | --- | --- | --- |
| BiP/GRP78 | STB | Increased protein expression in CTB and STB | (1) |
|  |  | Interacts with ⍺2-macroglobulin to regulate CTB fusion in BeWo cells | (2,3) |
|  |  | Regulates hCG secretion in BeWo cells | (3) |
|  | EVT | Regulates proinflammatory cytokines in HTR-8/SVneo cells | (1) |
|  | DSC | Highly expressed in glandular epithelium of decidua and decidualizing ESCs during early stages of placentation | (4–8) |
|  |  | Regulates prolactin and insulin-like growth factor-binding protein-1 expression | (9) |
|  | Placental tissue | Expressed at high levels during early gestation | (10) |
|  | Mice | BiP-null mice exhibit peri-implantation lethality | (11) |
|  | Pathology | Increased protein expression in spontaneous preterm birth | (1) |
|  |  | Circulating GRP78 can be used as a biomarker for preeclampsia | (12) |
| Calreticulin | STB | Calreticulin regulates hCG production and CTB fusion in BeWo cells | (13) |
|  | EVT | Calreticulin deficiency impairs EVT invasiveness by dysregulating synthesis of N-glycans. | (14) |
|  | DSC | Exchange protein directly activated by cAMP 2 regulates calreticulin expression in endometrial stromal and glandular epithelial cell-lines | (15,16) |
|  |  | Regulates prolactin and insulin-like growth factor-binding protein-1 expression in decidualizing human and rat endometrial stromal cells | (15,17) |
|  |  | Deficiency impairs *LIF* and *PTGS2* expression and secretion of PGE2 in endometrial glandular epithelial cells | (16) |
|  |  | Blocking calreticulin expression reduces embryo implantation in mouse models | (18) |
|  | Placental tissue | Calreticulin is expressed in STB, EVTs, and placental macrophages | (13) |
|  | Pathology | Maternal serums from preeclamptic pregnancies exhibit high levels of ER-stress induced calreticulin | (19) |
|  |  | Extracellular calreticulin impairs BeWo cell differentiation | (13) |
| Calnexin | STB | Calnexin deficiency can reduce hCG production and CTB fusion in BeWo cells | (20) |
| PDIs | EVT | PDIA3 deficiency increases apoptosis and decreases proliferation in HTR-8/SVneo cells | (21) |
|  | DSC | Six PDI isoforms expressed in endometrial epithelial cell-lines that model receptive and non-receptive endometrium. | (22) |
|  |  | PDI deficiency in implantation model using spheroids of JEG3 and AN3CA cell-lines enhanced endometrial receptivity | (22) |
|  | Placental tissue | *PDIA3* and *PDIA6* highly expressed in differentiated trophoblasts of first trimester placental tissue | (10) |
|  | Pathology | Reduced levels of PDIA3 in placental tissue from preeclamptic pregnancies, maternal obesity, and nicotine exposure. | (21,23,24) |

**References**

1. Tissarinen P, Tiensuu H, Haapalainen AM, Määttä TA, Ojaniemi M, Hallman M, et al. Elevated human placental heat shock protein 5 is associated with spontaneous preterm birth. Pediatr Res. 2023 Aug;94(2):520–9.

2. Bastida-Ruiz D, Wuillemin C, Pederencino A, Yaron M, Martinez De Tejada B, Pizzo SV, et al. Activated α2-macroglobulin binding to cell surface GRP78 induces trophoblastic cell fusion. Sci Rep. 2020 Jun 15;10(1):9666.

3. Fradet S, Pierredon S, Ribaux P, Epiney M, Shin Ya K, Irion O, et al. Involvement of Membrane GRP78 in Trophoblastic Cell Fusion. Oudejans C, editor. PLoS ONE. 2012 Aug 9;7(8):e40596.

4. Simmons DG, Kennedy TG. Induction of glucose-regulated protein 78 in rat uterine glandular epithelium during uterine sensitization for the decidual cell reaction. Biol Reprod. 2000 May;62(5):1168–76.

5. Lin P, Jin Y, Lan X, Yang Y, Chen F, Wang N, et al. GRP78 expression and regulation in the mouse uterus during embryo implantation. J Mol Histol. 2014 Jun;45(3):259–68.

6. Anelli T, Dalla Torre M, Borini E, Mangini E, Ulisse A, Semino C, et al. Profound architectural and functional readjustments of the secretory pathway in decidualization of endometrial stromal cells. Traffic. 2022;23(1):4–20.

7. Choi JY, Jo MW, Lee EY, Lee DY, Choi DS. Ovarian steroid dependence of endoplasmic reticulum stress involvement in endometrial cell apoptosis during the human endometrial cycle. 2018 Jun 1 [cited 2024 Sep 29]; Available from: https://rep.bioscientifica.com/view/journals/rep/155/6/REP-17-0713.xml

8. Brosens JJ, Salker MS, Teklenburg G, Nautiyal J, Salter S, Lucas ES, et al. Uterine Selection of Human Embryos at Implantation. Sci Rep. 2014 Feb 6;4:3894.

9. Fernández L, Kong CS, Alkhoury M, Tryfonos M, Brighton PJ, Rawlings TM, et al. The endoplasmic reticulum protein HSPA5/BiP is essential for decidual transformation of human endometrial stromal cells. Sci Rep. 2024 Oct 29;14(1):25992.

10. Prater M, Hamilton RS, Wa Yung H, Sharkey AM, Robson P, Abd Hamid NE, et al. RNA-Seq reveals changes in human placental metabolism, transport and endocrinology across the first–second trimester transition. Biology Open. 2021 Jun 15;10(6):bio058222.

11. Luo S, Mao C, Lee B, Lee AS. GRP78/BiP Is Required for Cell Proliferation and Protecting the Inner Cell Mass from Apoptosis during Early Mouse Embryonic Development. Molecular and Cellular Biology. 2006 Aug 1;26(15):5688–97.

12. Laverriere A, Landau R, Charvet I, Irion O, Bischof P, Morales M, et al. GRP78 as a marker of pre-eclampsia: an exploratory study. Molecular Human Reproduction. 2009 Sep 1;15(9):569–74.

13. Iwahashi N, Ikezaki M, Nishitsuji K, Yamamoto M, Matsuzaki I, Kato N, et al. Extracellularly Released Calreticulin Induced by Endoplasmic Reticulum Stress Impairs Syncytialization of Cytotrophoblast Model BeWo Cells. Cells. 2021 May 24;10(6):1305.

14. Yamamoto M, Ikezaki M, Toujima S, Iwahashi N, Mizoguchi M, Nanjo S, et al. Calreticulin Is Involved in Invasion of Human Extravillous Trophoblasts Through Functional Regulation of Integrin β1. Endocrinology. 2017 Nov 1;158(11):3874–89.

15. Kusama K, Yoshie M, Tamura K, Nakayama T, Nishi H, Isaka K, et al. The Role of Exchange Protein Directly Activated by Cyclic AMP 2-mediated Calreticulin Expression in the Decidualization of Human Endometrial Stromal Cells. Endocrinology. 2014 Jan 1;155(1):240–8.

16. Kusama K, Yoshie M, Tamura K, Imakawa K, Tachikawa E. EPAC2-mediated calreticulin regulates LIF and COX2 expression in human endometrial glandular cells. 2015 Feb 1 [cited 2024 Oct 30]; Available from: https://jme.bioscientifica.com/view/journals/jme/54/1/17.xml

17. Yoshie M, Kusama K, Tanaka R, Okubo T, Kojima J, Takaesu Y, et al. Possible Roles of Calreticulin in Uterine Decidualization and Receptivity in Rats and Humans. IJMS. 2021 Sep 29;22(19):10505.

18. Cheng SQ, He JL, Dong YL, Liu XQ, Ding YB, Gao RF, et al. Characterization of calreticulin expression in mouse endometrium during embryo implantation. Biol Res. 2009;42(4):505–16.

19. Gu VY, Wong MH, Stevenson JL, Crawford KE, Brennecke SP, Gude NM. Calreticulin in human pregnancy and pre-eclampsia. Molecular Human Reproduction. 2008 May 1;14(5):309–15.

20. Matsukawa H, Ikezaki M, Nishioka K, Iwahashi N, Fujimoto M, Nishitsuji K, et al. Calnexin Is Involved in Forskolin-Induced Syncytialization in Cytotrophoblast Model BeWo Cells. Biomolecules. 2022 Jul 28;12(8):1050.

21. Mo HQ, Tian FJ, Ma XL, Zhang YC, Zhang CX, Zeng WH, et al. PDIA3 regulates trophoblast apoptosis and proliferation in preeclampsia via the MDM2/p53 pathway. Reproduction. 2020 Aug;160(2):293–305.

22. Hannan NJ, Paiva P, Dimitriadis E, Salamonsen LA. Models for study of human embryo implantation: choice of cell lines? Biol Reprod. 2010 Feb;82(2):235–45.

23. Shen WB, Wang B, Yao R, Goetzinger KR, Wu S, Gao H, et al. Obesity impacts placental function through activation of p-IRE1a-XBP1s signaling. Front Cell Dev Biol. 2023 Feb 1;11:1023327.

24. Wong MK, Nicholson CJ, Holloway AC, Hardy DB. Maternal Nicotine Exposure Leads to Impaired Disulfide Bond Formation and Augmented Endoplasmic Reticulum Stress in the Rat Placenta. Jin DY, editor. PLoS ONE. 2015 Mar 26;10(3):e0122295.
